# Deciduous Teeth and the Evils of Their Decay and Loss

**Published:** 1888-06

**Authors:** Garrett Newkirk

**Affiliations:** Chicago.


ARTICLE V.

### DECIDUOUS TEETH AND THE EVILS OF THEIR DECAY AND LOSS.

BY DR. GARRETT NEWKIRK, CHICAGO.

[Read before the Dental Section of the American Medical Association.]

As the potter forms his beautiful designs in plastic clay, so nature forms all the organs of the body. At first they are soft, little more in appearance or consistency than so many bits of jelly. By slow degrees they are brought to the requisite density of foundation, framework and superstructure.

The harder the tissue, the longer the time of its development and the slower its changes. The permanent teeth are the hardest structures in the body. Because the permanent teeth are to be the hardest tissues of the body, they must have time for growth and maturity.

Formed first in the soft state their density depends on the slow deposit of lime salts, particle by particle. The process will require for its final completion nearly half a lifetime. A structure for a thousand years cannot be built in two years. The teeth of prehistoric men still remain in mounds where even bones have crumbled. To supply the wants of the child while these are building, nature has provided a deciduous set.

The law of existence as to the temporary tooth is this: Each has a legitimate successor elected to take his seat at a certain time. The first should remain till the time of the second. The deciduous central incisors should remain till the seventh year, the laterals till the eighth, the molars till the ninth or tenth, the cuspids, last of all, till the tenth or eleventh year. From the age of seven to eleven or twelve nature intends the child to have a mixt set of teeth. Whether it be true or not that she abhors a vucuum," she certainly despises a gap in a row of teeth. One office of the teeth, especially in early life, is to support one another. A row of teeth is something like a row of books—remove one, the others tend to fall together. Now mark this point: If a temporary tooth be prematurely lost while its successor is imperfectly calcified and unready, one of several undesirable things may happen: First, the tooth may fail to come down, then the space is lost by the falling together of the adjacent teeth, eruption may be thus retarded, or, as sometimes happened; entirely prevented; or, the teeth may erupt inside or outside the line of the arch, causing an irregularity; or, it may erupt too soon, imperfectly calcified, and so be readily attacked by decay.

Most of the cases of protruding cuspids so frequently seen are caused by the premature loss of the temporary. An exception is where the temporary cuspid is retained *too* long, producing the same result. The rule is that the temporary cuspid should remain in the mouth till the child is eleven years old, sometimes longer. It is safe to say that, till the temporary teeth are well in place, the indications of nature are plainly against the free use of solid food. When the teeth are in place, nature is ready for the introduction of a more substantial diet than milk.

The uses of the temporary teeth are the same as of those that follow. They were created because they were needed. The food of the child needs the facility for mastication. The premature loss of a tooth is a loss to mastication and digestion. The same material want which called for the *existence* of the tooth calls also for its use and *preservation*..

The troubles which proceed from diseased teeth in the adult follow from the same cause in children. Whatever causes nervous irritability in children we all know should be avoided. Very few people realize how much they suffer from neglected teeth. The eruption of the teeth is far less apt to produce reflex disturbance than their decay after eruption. Nothing is better calculated than a congested pulp to cause pain, insomnia, general nervousness, and irritability. Such disturbances continuing, indigestion, malnutrition and intestinal troubles follow. Convulsive tendencies are also increased. Within two years I have seen a case of chorea in a child ten years old, associated with diseased teeth. The filling of these, with protection to several nearly exposed pulps, was followed by speedy recovery. Since the attention of ophthalmologists and otologists has been given more to the cause and treatment of reflex nervous influence, it has been found that diseased teeth in children, as well as in adults, are often the real source of troubles referred to the eye and ear.

Parents usually neglect their duty to their children's teeth. They do it not wilfully, but through ignorance. The physician is supposed to know about them, but is apt to forget the teeth of his little patients. The dentist is not consulted till the mischief is done, and too often he lacks the definite knowledge, patience and skill to do the children justice. Decay is permitted to go on, associated with more or less irritation and reflex disturbance, till the pulp of the tooth dies. Then the putrefaction of the dead pulp tissue begins with the evolution of poisonous gases. If these do not find escape through the cavity of decay they pass through the end of the root into the investing tissues, causing inflammation and abscess. What people call a "gumboil" is an opening for the discharge of pus from an abscess connected with the root of a neglected tooth with a putrefying pulp. The pulp is discharged almost daily and mingles in the fluids of the mouth. It is mixed with the saliva and food and swallowed. It may or may not be poisonous; it certainly is not wholesome. The teeth thus affected are tender on pressure; that is, the tissues under the root are sensitive and painful when the tooth is touched. The swelling of their underlying membrane makes them sometimes protrude a little when the child attempts to bring his teeth together in mastication. The sore ones touch first; therefore, he avoids chewing, and swallows his food as best he can. He is likely to contract the habit of bolting his food, and this habit may cling to him long after the existing cause has been removed.—*Items of Interest.*

